# Efficacy of Sesquichloride of Iron for the Treatment of Ulcers about the Nails

**Published:** 1863-09

**Authors:** 


### EFFICACY OF SESQUICHLORIDE OF IRON FOR THE TREATMENT OF ULCERS ABOUT THE NAILS.

M. Billon communicates the following to the *Journal de Med. et de Chirurgie :*

Dr. Caillet of Luynes (Indre et Loire,) having recently published a case in which the application of sesquichloride of iron effected a cure of the affection popularly termed *the growth of the nail into the flesh*, I take this opportunity of recording several instances of the same kind, witnessed by myself, which confirm the results obtained by M. Caillet, and may perhaps be deemed not wholly uninteresting. In 1858, Dr. Wahu, staff-physician to the army, having succeeded with this remedy in curing the painful diseases in question, I resorted to the same method, and with the greatest benefit in four cases. I may here remark that ulcers about the nails are occasionally observed among our soldiers, having escaped the attention of the medical boards, or being caused by the pressure of the boot during forced marches. Under these circumstances, a *prompt* and *painless* cure may be effected by inserting the *dry* sesquichloride between the nail and the protruding flesh, and powdering the latter with the same substance. A large bandage should be applied over all, not impregnated with the *liquid* sesquichloride of iron, as recommended by Dr. Caillet, a precaution which may, however, be useful, as the folds of the band dry rapidly, and preserve their situation in a more exact manner.

On the following day, the exuberant flesh is found to have acquired the hardness of wood; suppuration speedily ceases, and a cure follows after two or three applications. This simple and mild treatment is obviously far preferable to the numerous surgical procedures hitherto recommended. In the course of four or five days, in a week at the farthest, the original pain ceases, the swelling subsides, and the patient is able to walk. Naught remains but the hardened protruding flesh, which falls away about a month after the application of the sesquichloride of iron. These are the results yielded by the method in four soldiers suffering from the growth of the nail into the flesh. They have appeared to be sufficiently remarkable to warrant the communication.—*Reporter*.

